# Items of General Interest

**Published:** 1915-06

**Authors:** 


### Items of General Interest.

In a recent issue of the JOURNAL we published an article concerning the organization of the "Texas Surgical Society," which is composed of the following officers and charter members:

President, James E. Thompson; first vice president, J. H. Reuss; second vice president, F. B. Paschal; secretary, W. Burton Thorning: treasurer, J. B. Smoot; recorder, I. C. Chase; council, John T. Moore, H. M. Doolittle, H. R. Dudgeon; committee on semi-annual meeting, R. R. White, F. C. Beall.

Charter Members—K. H. Aynesworth, Waco; F. L. Barnes, Houston; F. C. Beall, Fort Worth; Joe Becton, Greenville; I. C. Chase, Fort Worth; H. M. Doolittle, Dallas; H. R. Dudgeon, Waco; J. W. Hale, Waco; W. G. Jameson, Palestine; John T. Moore, Houston; F. B. Paschal, San Antonio; E. J. Reeves, Dallas; J. H. Reuss, Cuero; Bacon Saunders, Fort Worth; A. C. Scott, Temple; A. B. Small, Dallas; J. B. Smoot, Dallas; J. E. Thompson, Galveston; W. Burton Thorning, Houston; C. S. Venable San Antonio; R. R. White, Temple.

We have just received the constitution and by-laws of this society and we feel that the article on membership will prove of especial interest to those who are seeking admission into this distinguished organization.

### ARTICLE III.

### MEMBERSHIP.

Section 1. This Society shall consist of Active and Honorary Fellows.

Sec. 2. The active membership shall be limited to one hundred and twenty-five (125) Fellows, who shall be residents of the State of Texas. The honorary membership shall be limited to twenty-five (25) Fellows.

Sec. 3. The charter members of this Society shall be limited to those men who are limiting their work to surgery.

Sec. 4. The selection of future candidates for membership shall be made by the Council and the names shall be presented at a general meeting of the Fellows for acceptance or rejection.

Sec. 5. If objection be raised to any candidate, the name must be voted upon by all members present and must receive seventy-five (75) per cent of the votes cast.

Sec. 6. Propositions for Fellowship shall be made by Fellows on blanks furnished by the Secretary of the Society. The proposal of a candidate for Fellowship shall be signed by three Fellows, who shall vouch for his character and standing. No one shall be eligible for Fellowship unless he is at least thirty years of age and a graduate of five years standing from a recognized medical college, and has established a reputation as a practitioner, author, teacher or original investigator, and has been recommended by the Council. All propositions for Fellowship shall lie over for six months from the time of their reception by the Society.

Sec. 7. The proposers of a candidate shall, within thirty days of the reception of the proposal for Fellowship, send to the Secretary a confidential letter, an abstract of which, without the name of the writer, shall be presented by the Secretary to the Council, and the signature returned to the writer.

Sec. 8. Fellows after election must qualify within three months by the payment of the initiation fee and annual dues.

Sec. 9. Any candidate for Fellowship rejected by the Society cannot be proposed again for membership except after the lapse of two years.

Sec. 10. Honorary Fellows shall be proposed and elected in the same manner as active Fellows. They shall not be required to pay any fees or dues, nor shall they be privileged to vote or hold office.

Sec. 11. The resignation of a Fellow may be accepted by a two-thirds vote of the members present, provided he is not in arrears or under charges pending.

Sec. 12. Any violation of the Constitution or By-Laws, or conduct unbecoming a gentleman, may be punished by a reprimand, suspension or expulsion.

Sec. 13. Any active Fellow who shall absent himself without presenting valid reasons, from three consecutive meetings shall be dropped from Fellowship in the Society. He shall then be notified of the fact by the Secretary, and can only be reinstated by unanimous vote of the Council.

Sec. 14. Every candidate for Fellowship shall be required to sign a pledge binding himself not to indulge in fee-splitting in any way, shape or form.

Below is appended the Fellowship pledge, which every man who joins the Association must take.

FELLOWSHIP PLEDGE.

Recognizing that the Texas Surgical Society seeks to exemplify, enforce and develop the highest traditions of our calling, I hereby pledge myself, as a condition of Fellowship in the Society, to live in strict accordance with all its principles, declarations and regulations.

In particular I pledge myself to pursue the practice of surgery with thorough self-restraint and to place the welfare of my patients above all else; to advance constantly in knowledge by the study of surgical literature, the instruction of eminent teachers, interchange of opinion among associates, and attendance on the important societies and clinics; to regard scrupulously the interests of my professional brothers and seek their counsel when in doubt of my own judgment, to render willing help to my colleagues and to give freely my services to the needy.

Moreover, I pledge myself, so far as I am able, to avoid the sins of selfishness; to shun unwarranted publicity, dishonest money seeking and commercialism as disgraceful to our profession; to teach the patient his financial duty to the physician and to urge the practitioner to obtain his reward from the patient openly; to make my fees commensurate with the services rendered and with the patient's rights; and to avoid discrediting my associates by taking unwarranted compensation.

I further pledge myself upon my honor as a gentleman that I will not, so long as I am a member of the Texas Surgical Society, or at any future time, practice division of fees in any form; nor will I fix, or attempt to fix, the charges of other physicians who may be associated with me in any case; neither will I collect fees for other physicians referring patients to me when by such a collection the collector shall retain any part of the total amount of money collected, either as a professional fee or as a commission for such collection. I will not make joint fees with physicians or surgeons to whom I may refer patients, or who may refer patients to me; nor will I make joint fees in cases in which I am associated and sharing responsibility with another physician or surgeon; neither will I, in any way, either directly or indirectly, compensate or accept compensation for any case referred by me or to me.

Finally, I pledge myself to co-operate in advancing and extending, by every lawful means within my power, the influence of the Texas Surgical Society.

Signature........................

---

The Southern Sociological Congress which met recently in Houston in three days session was largely attended by prominent men from all over the United States. It is impossible to mention even in a general way the varied topics which were discussed by men who are leaders and authority on the subject upon which they spoke. Our own Dr. S. P. Brooks of Waco was elected president of this Congress for the coming year, and it is to be hoped that every physician in Texas will co-operate with him to the extent of his ability in this crusade for better health.

The following was given out as the creed and the crusade of the Southern Sociological Congress:

We believe—

1. That God our Father is the giver of all life.
2. That health is life as it ought to be.
3. That health is the basis of prosperity and happiness, and therefore our first duty both individually and socially.
4. That 50 per cent of the deaths in our country are preventable, and that 90 per cent of communicable diseases should be prevented.
5. That the essential and first work of the medical profession is the conservation, not the correction of health, and that the physician should be paid for preventing disease, rather than for curing it.
6. That the Federal government should establish a co-ordinate cabinet department of health.

7. That the death of children is a defeat of God's purpose, and their health—physical, mental and moral—should be a primary function and responsibility of the church.

8. That the promotion of the health of children and of the community should be to a school of corresponding interest and obligation with instruction.

9. That the press can render at the present time no greater service to the nation than to champion the cause of public health.

10. That the time has come for a nation-wide crusade for health. And we call—

On the people of the South to co-operate, through the agencies of home and school, medical profession and press, church and government, for the achievement of health for the individual, for the community and for the nation.

One of the most important matters brought before the house of delegates at the recent State Medical Association held in Fort Worth came from Dr. L. B. Bibb of Austin in the shape of a committee report on the study of cottonseed meal as a food for humans. While the report tended to show the experimental stage, Dr. Bibb said Miss Anna D. Richardson of the domestic science department of the State University with the aid of experts from Columbia University is working on the problem.

Dr. J. H. Lander of Greenville has purchased the interest of his partner, Dr. R. M. Prather, in the Beeville hospital; the same will be open to the use of the medical profession.

A complete hospital unit, composed of thirty-two Chicago doctors and seventy-four nurses, under the direction of Dr. John B. Murphy, Chicago, will be Chicago's contribution to the British army.

Dr. W. C. Rucker, Assistant Surgeon General, Washington, D. C., declares that measles is more dangerous than plague, because care is not given it and complications follow which cause death.

We have before us Vol. 1, No. 4 of the *Southwestern Hospital Reporter*. The JOURNAL is interesting and helpful and it is high grade in every sense of the word. We wish for Dr. Wm. R. Helie, the owner and publisher the success such a worthy publication deserves. Write Dr. Helie, Houston, for sample copy.

Dr. Lawrence J. Rhea, a native of Collin county, Texas, graduate of the State University, 1901, later of Johns Hopkins Medical School, has become a captain in the medical corps of the British army.

Dr. Harry Plotz, a young bacteriologist of Mount Sinai Hospital, New York, "the Man of the Hour" in the New York medical circles today, announces that he has discovered an anti-typhus vaccine. In view of the recent spread of this dread disease in Serbia, where many American physicians are leading in the fight against it, the discovery was hailed by physicians as timely as well as important.

The National Association for the Study and Prevention of Tuberculosis has inaugurated a movement for the purpose of securing more co-operation from physicians and nurses in the anti-tuberculosis campaign. "The object of this campaign," says Dr. Charles J. Hatfield, executive secretary, is: "Primarily to secure more accurate and earlier diagnosis of tuberculosis on the part of physicians and to show nurses the great opportunities of service in the home care of consumptives."

The American Proctologic Society will hold its seventeenth annual meeting in San Francisco, Cal., June 21-22, 1915, with headquarters at the St. Francis Hotel, Civic Auditorium. The profession is cordially invited to attend all the meetings.

Dr. Otto V. Huffman, secretary of the New York State Board of Medical Examiners, has been elected secretary of the faculty and executive head of Long Island College Hospital, Brooklyn, in succession to the late Dr. Joseph H. Raymond. Dr. Huffman is a native of Dayton, Ohio, is 33 years old and is a graduate of Denison University and the College of Physicians and Surgeons of Columbia University, 1903.

Those who contemplate attending the American Medical Association will be glad to know that there are three special plans which may be patronized, viz: First, the Gregory tour; second, the McCann tour, and, third, the Pennsylvania Railroad tours. By writing to Gregory Tours Co., Lytton Building, Chicago, all information wanted can be obtained. The tour system of the Pennsylvania Railroad is being operated in the interest of the Pan-American Medical Congress, which meets in San Francisco, June 17-21, also the American Medical Association meeting, which follows immediately thereafter. For additional information and booking on either the Pan-American Medical Congress, special of the American Medical Association special, application should be made to Dr. H. L. E. Johnson, chairman transportation committee, Pan-American Medical Congress, 1821 Jefferson Place N. W., Washington, D. C. For information regarding the McCann tours, write to J. Rawson Pennington, M. D., Columbus Memorial Building, Chicago, chairman committee on transportation and place of session. All information regarding tickets, Pullman, rooms at the hotel and places of amusement on the exposition grounds may be obtained by writing for same.

---

### The Seventh Pan-American Congress.

The seventh Pan-American Congress will meet in San Francisco, June 17-21, inclusive. It assembles pursuant to invitation of the President of the United States issued in accordance with an act of Congress approved March 3, 1915.

The countries and colonies embraced in the Congress are the Argentine Republic, Bolivia, Brazil, Canada, Colombia, Cuba, Chile, Costa Rica, El Salvador, Ecuador, Guatamala, Honduras, Haiti, Hawaii, Mexico, Martinique, Nicaragua, Panama, Paraguay, Peru, Santo Domingo, United States, Uruguay, Venezuela, British Guiana, Dutch Guiana, French Guiana, Jamaica, Barbadoes, St. Thomas and St. Vincent. The organization of the Congress is perfected in these countries and the majority of them have signified their intention to be represented by duly accredited delegates.

The Congress will meet in seven sections, viz.: (1) Medicine; (2) Surgery; (3) Obstetrics and Gynecology; (4) Anatomy, Physiology, Pathology and Bacteriology; (5) Tropical Medicine and General Sanitation; (6) Laryngology, Rhinology and Otology; (7) Medical Literature.

All members of the organized medical profession of the constituent countries are eligible and are invited to become members. The membership fee is $5.00 and entitles the holder to a complete set of the transactions. Advance registrations are solicited and should be sent with membership fee to the Treasurer, Dr. Henry P. Newman, Timken Building, San Diego, California.

